# Whole-Genome Sequence of Quorum-Sensing Vibrio tubiashii Strain T33

**DOI:** 10.1128/genomeA.01362-14

**Published:** 2015-01-02

**Authors:** Nur Izzati Mohamad, Wai-Fong Yin, Kok-Gan Chan

**Affiliations:** Division of Genetics and Molecular Biology, Institute of Biological Sciences, Faculty of Science, University of Malaya, Kuala Lumpur, Malaysia

## Abstract

Vibrio tubiashii strain T33 was isolated from the coastal waters of Morib, Malaysia, and was shown to possess quorum-sensing activity similar to that of its famous relative Vibrio fischeri. Here, the assembly and annotation of its genome are presented.

## GENOME ANNOUNCEMENT

In 1965, Tubiash, Chanley, and Leifson (1) first discovered pathogenic bacteria that infect the mollusk, and these were classified as Vibrio anguillarum. However, due to the advancement of molecular taxonomical approaches, further work done in 1984 by Hada and colleagues (2) confirmed that the bacterium isolated in 1965 by Tubiash, Chanley, and Leifson is a new species, which was named Vibrio tubiashii. Isolated in 2014, V. tubiashii strain T33 forms a round yellow colony on Luria-Bertani agar (LBA) with 3% (wt/vol) NaCl concentration. Here, we sequenced the whole genome of V. tubiashii strain T33, as this will contribute to the understanding of its pathogenic properties and pave the way to solutions to combat severe vibriosis in mollusks, such as clams, oysters, and shellfish.

By using the QIAamp DNA minikit (Qiagen, Germany), the genomic DNA of V. tubiashii strain T33 was isolated according to the manufacturer’s recommendations. DNA quality was checked via a NanoDrop spectrophotometer (Thermo Scientific) and Qubit 2.0 fluorometer (Life Technologies). Using the platform Illumina MiSeq personal sequencer (Illumina, Inc., CA), the whole genome of V. tubiashii strain T33 was sequenced. The number of calculated filtered reads was 1,308,193, with approximately 60.05-fold coverage. Assembly of the filtered reads was done using CLC Genomics Workbench version 5.1 (CLC bio, Denmark) (3) and resulted in 46 contig numbers, with an *N*_50_ value of 410,629. A total of 4,144,653 bp makes up the draft genome of V. tubiashii strain T33, with a G+C content of 45%. Annotation was done using the Rapid Annotations using Subsystems Technology (RAST) server (4). The number of open reading frames (ORFs) of strain T33 is 3,815, and the number of tRNAs predicted using tRNAscan-SE (version 1.21) (5) is 76. There are 5 main housekeeping genes in strain T33, which consist of three copies of 5S rRNA genes, one copy of 23S rRNA gene, and one copy of 16S rRNA gene, which was characterized using RNAmmer (6).

From the RAST server annotation, we discovered a protein that was responsible for the *N*-acyl homoserine lactone (AHL), LuxM, in contig number 8. AHL is a quorum-sensing (QS) signaling molecule that has been reported in many proteobacteria, including marine vibrios (7, 8). LuxM is a homologue to LuxI, which is a protein responsible for the production of signal molecules, namely, AHLs (9, 10). The QS signaling system, which consists of genes and regulators, may play a key role in the pathogenic properties of V. tubiashii. Therefore, this study may provide insight to better understand the virulence factors of V. tubiashii, hence leading to an effective solution for severe vibriosis.

### Nucleotide sequence accession number.

This whole-genome shotgun project has been deposited at DDBJ/EMBL/GenBank under the accession no. JRWQ00000000. The version described in this paper is the first version.
